# C-reactive protein is not a useful indicator for infection in surgical intensive care units

**DOI:** 10.1590/S1516-31802009000600006

**Published:** 2010-05-21

**Authors:** Domingos Dias Cicarelli, Joaquim Edson Vieira, Fábio Ely Martins Benseñor

**Affiliations:** I MD, PhD. Attending anesthesiologist, Intensive Care Unit, Anesthesia Division, Department of Surgery, Hospital das Clínicas da Faculdade (HC), Faculdade de Medicina da Universidade de São Paulo (FMUSP), São Paulo, Brazil.; II MD, PhD. Attending anesthesiologist, Anesthesia Division, Department of Surgery, Hospital das Clínicas (HC), Faculdade de Medicina da Universidade de São Paulo (FMUSP), São Paulo, Brazil.

**Keywords:** Shock, septic, C-reactive protein, Systemic inflammatory response syndrome, Multiple organ failure., Lactates, Choque séptico, Proteína C-reativa, Síndrome de resposta inflamatória sistêmica, Insuficiência de múltiplos órgãos, Lactatos

## Abstract

**CONTEXT AND OBJECTIVE:**

C-reactive protein (CRP) is commonly used as a marker for inflammatory states and for early identification of infection. This study aimed to investigate CRP as a marker for infection in patients with postoperative septic shock.

**DESIGN AND SETTING:**

Prospective, single-center study, developed in a surgical intensive care unit at Hospital das Clínicas, Faculdade de Medicina da Universidade de São Paulo.

**METHODS:**

This study evaluated 54 patients in the postoperative period, of whom 29 had septic shock (SS group) and 25 had systemic inflammatory response syndrome (SIRS group). All of the patients were monitored over a seven-day period using the Sequential Organ Failure Assessment (SOFA) score and daily CRP and lactate measurements.

**RESULTS:**

The daily CRP measurements did not differ between the groups. There was no correlation between CRP and lactate levels and the SOFA score in the groups. We observed that the plasma CRP concentrations were high in almost all of the patients. The patients presented an inflammatory state postoperatively in response to surgical aggression. This could explain the elevated CRP measurements, regardless of whether the patient was infected or not.

**CONCLUSIONS:**

This study did not show any correlation between CRP and infection among patients with SIRS and septic shock during the early postoperative period.

## RESUMO

**CONTEXTO E OBJETIVO:**

A proteína C reativa (PCR) é muito usada como marcador de estados inflamatórios e na identificação precoce de infecção. Este estudo teve como proposta investigar a PCR como marcadora de infecção em pacientes em choque séptico no período pós-operatório.

**TIPO DE ESTUDO E LOCAL:**

Estudo prospectivo, monocêntrico, desenvolvido numa unidade de terapia intensiva pós-operatória do Hospital das Clínicas da Faculdade de Medicina da Universidade de São Paulo.

**MÉTODOS:**

Foram avaliados 54 pacientes no pós-operatório, sendo 29 deles com choque séptico (grupo SS) e 25 com síndrome da resposta inflamatória sistêmica (grupo SI). Todos os pacientes foram acompanhados durante sete dias pelo escore SOFA (Sequential Organ Failure Assessment) e com dosagens diárias de PCR e lactato.

**RESULTADOS:**

As dosagens de PCR não diferiram entre os grupos. Não foi observada correlação entre dosagem de PCR e lactato ou escore SOFA nos grupos estudados. Observamos que as concentrações plasmáticas de PCR estavam elevadas em quase todos os pacientes avaliados. Os pacientes no pós-operatório apresentam estado inflamatório em resposta à agressão cirúrgica, sendo este fato capaz de explicar as dosagens de PCR elevadas, independentemente de o paciente estar ou não infectado.

**CONCLUSÕES:**

Este estudo não evidenciou correlação entre PCR e infecção nos pacientes com síndrome da resposta inflamatória sistêmica e choque séptico no período pós-operatório precoce.

## INTRODUCTION

Systemic inflammatory response syndrome (SIRS) is a common event among critically ill patients. Whether accompanied by infection or not, it is frequent postoperatively, particularly in relation to trauma, burns, pancreatitis or pulmonary diseases.^1^ Conversely, severe sepsis and septic shock are states of systemic inflammation in response to infectious agents that may lead to multiple organ system failure, and these are a frequent cause of postoperative mortality in intensive care units (ICUs).^2^

The release of inflammatory response mediators over the course of such diseases gives physicians a useful tool for marking the severity of sepsis.^3^ One of these markers, C-reactive protein (CRP), is produced by the liver in response to tissue injury or infection. It reaches its maximum serum concentration around 24 hours after the inflammatory process sets in and slowly decreases thereafter.^4^ Previous studies have presented doubtful conclusions regarding the efficiency of CRP for differentiating infection from inflammation, especially during the postoperative period.^5^^,^^6^

## OBJECTIVE

Considering the importance of distinguishing inflammatory septic response from non-infective events, this study had the aim of evaluating the role of CRP as a marker for infection in critically ill patients during the postoperative period.

## METHODS

This prospective study was performed in the surgical ICU at Hospital das Clínicas (HC), Faculdade de Medicina de São Paulo (FMUSP). After approval by the local ethics committee, patients admitted to the ICU with a diagnosis of SIRS or septic shock (SS) in accordance with the definitions of the American College of Chest Physicians/Society of Critical Care Medicine Consensus Conference were included.^7^ Informed consent was obtained from the patients or from their next of kin.^8^ Patients under 18 years of age were excluded.

At the time when patients were admitted into the study, their severity of illness was assessed according to the Acute Physiology And Chronic Health Evaluation II score (APACHE II).^9^ They were also assessed daily by using the Sequential Organ Failure Assessment score (SOFA) for seven consecutive days or until their discharge from the ICU, if this occurred earlier.^10^^,^^11^ C-reactive protein was measured on a daily basis.

For infection to be diagnosed, clinical signs of SIRS and a defined source of microbiologically confirmed infection (surgical finding) or positive blood, urine, catheter tip or tracheal secretion cultures had to be present.^5^

The patients received conventional therapy regarding antibiotic regimens, serial blood cultures and discharge criteria. The relevant clinical and laboratory tests were conducted daily throughout the study.

Blood samples for CRP measurements were thawed and assayed in batches in an automated analyzer (Behring Nephelometer Analyzer II, Dade Behring, Marburg, Denmark) for particle-enhanced immunonephelometry using commercial kits. The analytical sensitivity and accuracy of the CRP assays was 0.0175 mg/l (coefficient of variation, CV = 7.6%).

Statistical analysis to evaluate changes in variables over the course of the ICU stay was performed by means of two-way analysis of variance (ANOVA). Student’s t-test was used to analyze differences between groups. P < 0.05 was considered significant. The sample size was calculated as 25 patients per group, based on the standard normal deviation for a = 0.05 and b = 0.20.^12^

## RESULTS

**Figure 1.**
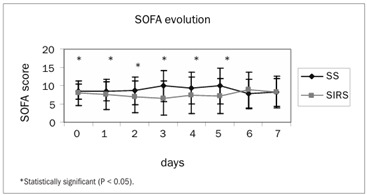
Daily evolution of sequential organ failure assessment (SOFA) score among the SS (septic shock) and SIRS (systemic inflammatory response syndrome) groups.

**Figure 2.**
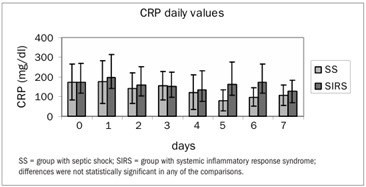
Mean C-reactive protein (CRP) measurements in the two groups.

**Figure 3.**
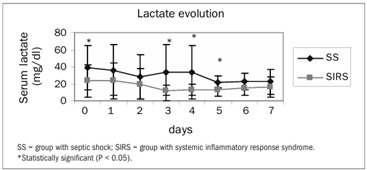
Mean serum lactate measurements in the two groups.

Out of the 59 patients enrolled, 29 formed the SS group and 25 formed the SIRS group. Five patients were excluded after their next of kin withdrew their signed consent. The patients’ characteristics at the time of admission to the study, their prior or preexisting conditions, the type of surgery and the outcome data are presented in Table 1. The microbiological characteristics of both groups are presented in Table 2. The evolution of SOFA scores in the two groups is presented in Figure 1. The daily evolution of CRP in the two groups is presented in Figure 2. Serum lactate was different between groups, as shown in Figure 3.

There was no correlation between CRP and SOFA in either group (r = 0.004; P = 0.99). There was a positive correlation between CRP and lactate in both groups, but without statistical significance (r = 0.60; P = 0.15).

**Figure 4.**
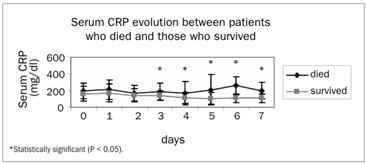
Outcome prognosis according to C-reactive protein (CRP) levels.

The daily evolution of CRP plasma concentrations among patients who died and survivors is presented in Figure 4. The mortality rate over seven days was 38% for the SS group (11 out of 29 patients) and 24% for the SIRS group (six out of 25 patients) (P = 0.28). Over 28 days, the mortality rates in the SS and SIRS groups were, respectively, 62% (18 out of 29) and 44% (11 out of 25) (P = 0.17). The SS group presented a seven-day relative risk (RR) of mortality of 1.6 (95% confidence interval, CI: 0.99-2.59), in comparison with the SIRS group; the 28-day relative risk of mortality was 1.4 (95% CI: 0.83-2.35).

**Table 1.** Patient characteristics in the two groups, at the time of admission to the study

Group	SS	SIRS	P
Characteristics	n = 29	n = 25	
Age (years)	59.4 ± 16.4	57.2 ± 19.1	NS
Male sex	55%	56%	NS
Weight (kg)	67.5 ± 13	67.3 ± 10.8	NS
APACHE II score	19 ± 5	16 ± 5	0.02
SOFA score	8.4 ± 3.7	8.1 ± 4.4	0.01
Preexisting conditions	n (%)	n (%)	
Hypertension	10 (34)	7 (28)	NS
Myocardial infarction	5 (17)	3 (12)	NS
Diabetes	4 (14)	4 (16)	NS
Liver disease	3 (10)	1 (4)	NS
COPD	2 (7)	2 (8)	NS
Cancer	6 (21)	5 (20)	NS
Surgery			
Multiple trauma (excluding head trauma)	1 (3.4)	1 (4)	NS
Gastrointestinal surgery	21 (72)	15 (60)	NS
Abdominal aneurysm repair	2 (7)	2 (8)	NS
Thoracic surgery	1 (3.4)	1 (4)	NS
Urologic surgery	3 (10)	2 (8)	NS
Other indicators of severity (days)	Mean ± SD	Mean ± SD	
Mechanical ventilation	4.4 ± 2.6	3.2 ± 2.3	NS
Shock (use of vasopressor)	3.7 ± 2.4	2.3 ± 2.2	0.03

SS = group with septic shock; SIRS = group with systemic inflammatory response syndrome; APACHE = acute physiology and chronic health evaluation; SOFA = sequential organ failure assessment; COPD = chronic obstructive pulmonary disease; NS = not statistically significant; SD = standard deviation.

**Table 2.** Microbiological analysis on patients in the two groups, including surgical procedure performed, antibiotic therapy, etiological infectious agent and source from which the agent was isolated

Patient	Surgery/pathological condition	Antibiotics	Type of organism	Type of culture
1-SS	Cholecystectomy/biliary abscess	Vancomycin + cefepime	*S. aureus*	Abscess culture
2-SS	Empyema pleural drainage	Ceftriaxone + clindamycin	*S. pyogenes*	Pleural abscess culture
3-SS	Cholecystectomy/biliary abscess	Ceftriaxone + metronidazole	-	Negative cultures
4-SS	Cystectomy/pyuria	Ceftriaxone + metronidazole	-	Negative cultures
5-SS	Abdominal aneurysm repair	Ceftazidime + clindamycin	*P. aeruginosa*	Blood culture
6-SS	Colectomy/cavity contamination	Ceftriaxone + metronidazole	-	Negative cultures
7-SS	Calcaneal exposure fracture	Ciprofloxacin	*E. faecalis*	Surgical site culture
8-SS	Pyonephrosis drainage	Ceftriaxone	*K. pneumoniae*	Urinary culture
9-SS	Sigmoidectomy	Ceftriaxone + metronidazole	*A. baumanii*	Blood culture
10-SS	Hemicolectomy	Ceftriaxone + metronidazole	*Candida albicans*	Blood culture
11-SS	Enterectomy/mesenteric ischemia	Ceftriaxone + metronidazole	-	Negative cultures
12-SS	Pancreatic-duodenal resection	Ceftriaxone	*Serratia marcesens*	BAL
13-SS	Pancreatic-duodenal resection	Ceftriaxone + metronidazole	*S*. coag negative	Blood culture
14-SS	Retroperitoneal abscess drainage	Cefepime + vancomycin + imipenem	*P. aeruginosa*	Blood culture
15-SS	Abdominal aneurysm repair	Vancomycin + imipenem	*S. aureus*	Blood culture
16-SS	Sigmoidectomy/perforative lesion	Ceftriaxone + metronidazole	*Serratia marcesens*	Ascites culture
17-SS	Colectomy	Cefepime + vancomycin	*S. aureus*	Blood culture
18-SS	Gastric ulcer	Ceftriaxone + metronidazole	-	Negative cultures
19-SS	Cholecystectomy	Ciprofloxacin + metronidazole	*Escherichia coli*	Urinary culture
20-SS	Hemicolectomy	Cefepime + vancomycin + metronidazole	*E. cloacae*	Blood culture
21-SS	Enterectomy/cavity contamination	Vancomycin + imipenem	-	Negative cultures
22-SS	Colectomy	Ceftriaxone + metronidazole	*A. baumanii*	Blood culture
23-SS	Colectomy	Ceftriaxone + metronidazole	*P. aeruginosa*	Blood culture
24-SS	Enterectomy/cavity contamination	Ceftriaxone + metronidazole	-	Negative cultures
25-SS	Cervical abscess drainage	Imipenem + vancomycin + metronidazole	*K. pneumoniae*	Blood culture
26-SS	Sigmoidectomy/perforative lesion	Ceftriaxone + metronidazole	*P. aeruginosa*	Blood culture
27-SS	Sigmoidectomy	Cefepime + metronidazole	*S. aureus*	Blood culture
28-SS	Pyonephrosis drainage	Cefepime + metronidazole	-	Negative cultures
29-SS	Colectomy	Ceftriaxone + metronidazole	*P. aeruginosa*	BAL
1-SIRS	Pancreatic-duodenal resection	Cephalothin	_	Blood culture
2-SIRS	Aortic-iliac bypass	Clindamycin	_	Blood culture
3-SIRS	Hemicolectomy	Cefoxitin	_	_
4-SIRS	Nephrectomy	Cephalotin	_	Blood and urinary
5-SIRS	Gastric resection	Cefoxitin	_	Blood culture
6-SIRS	Femoral exposure fracture	Clindamycin + gentamicin	_	Surgical site culture
7-SIRS	Abdominal aneurysm repair	Cephalothin	_	Blood and urinary
8-SIRS	Appendectomy	Cephalothin	_	Blood culture
9-SIRS	Hemicolectomy	Cefoxitin	_	Blood and urinary
10-SIRS	Prostatectomy	Cefazolin	_	Blood culture
11-SIRS	Colectomy	Cefoxitin	_	_
12-SIRS	Iliac endarterectomy	Cefazolin	_	Blood and urinary
13-SIRS	Colectomy	Cefoxitin	_	Blood culture
14-SIRS	Cholecystectomy	Cefazolin	_	_
15-SIRS	Colectomy	Cefoxitin	_	Blood culture
16-SIRS	Cholecystectomy	Cephalothin	_	_
17-SIRS	Hemicolectomy	Cefoxitin	_	Blood and urinary
18-SIRS	Lobectomy	Cefazolin	_	Blood culture
19-SIRS	Sigmoidectomy	Ceftriaxone + metronidazole	_	Blood culture
20-SIRS	Ulnar exposure fracture	Clindamycin + gentamicin	_	Surgical site culture
21-SIRS	Femoral exposure fracture	Clindamycin + gentamicin	_	Surgical site culture
22-SIRS	Splenectomy	Cefazolin	_	_
23-SIRS	Abdominal aneurysm repair	Cefazolin	_	Blood and urinary
24-SIRS	Enterectomy	Cefoxitin	_	Blood culture
25-SIRS	Cholecystectomy	Cefazolin	_	Blood culture

SS = group with septic shock; SIRS = group with systemic inflammatory response syndrome; *S. aureus* = *Staphylococcus aureus*, *S. pyogenes* = *Streptococcus pyogenes*, *P. aeruginosa* = *Pseudomonas aeruginosa*, *E. faecalis* = *Enterobacter faecalis*, *K. pneumoniae* = *Klebsiella pneumoniae*, *A. baumanii* = *Acinetobacter baumanii*, *S*. coag negative = *Staphylococcus* coagulase negative; *E. cloacae* = *Enterobacter cloacae*; BAL = bronchoalveolar lavage.

## DISCUSSION

The present study did not show any correlation between CRP and diagnoses of infection among postoperative patients with SIRS and septic shock. Nor did the SOFA score correlate with CRP measurements.

The patterns of cytokine production differ for different inflammatory conditions, as does the acute-phase response. Acute-phase changes reflect the presence and intensity of inflammation. Therefore, they have been used as a clinical guide for diagnosis and care. Conversely, serum CRP has been widely used as a marker for inflammation and tissue injury, as well as for diagnostic purposes, thereby differentiating inflammatory and infectious diseases.^13^ Previous reports have taken the view that elevated CRP levels are also associated with adverse outcomes in healthy individuals, as well as in patients with stable angina or acute coronary syndromes.^14^

Among patients with plasma CRP concentrations higher than 10 mg/dl, 80 to 85% have bacterial infections, according to previous studies.^15^^,^^16^ In this present study, patients from both groups presented plasma CRP concentrations higher than 10 mg/dl during the seven-day observation period, but no difference between septic shock (infected) and SIRS (non-infected) patients was found, thus contrasting with the results of Gabay et al.^16^ It is important to stress that Meisner et al. observed that CRP plasma concentrations were postoperatively elevated in almost all patients, regardless of the type of surgery.^6^ Suprin et al. observed that CRP was not a valuable indicator of infection in a medical ICU, due to its poor sensitivity and specificity.^15^ Ugarte et al. reported that CRP levels were not much higher in infected patients with shock,^17^ and this was confirmed by the findings presented here. All the patients studied were in the postoperative period. This fact, also according to Meisner, could in itself explain the elevated CRP measurements observed, regardless of whether infection was present or not. Hence, CRP cannot be used as a marker for infection in the early postoperative period.

Higher CRP levels were observed among the patients in the SIRS group than among those in the SS group. This can be explained by the antibiotic regimens among the septic shock patients. Many patients with SIRS received only prophylactic antibiotic therapy, while septic shock patients received antibiotics during their entire ICU stay. Given that Ventetuolo et al. reported that CRP might be used to follow the response to antibiotic therapy,^18^ the septic patients’ response to the antibiotic regimen could be the reason for their lower CRP levels.

The difference in CRP concentrations between patients who survived and those who did not agrees with previous reports in which this protein was considered to be a valuable prognostic marker for death among patients with septic shock.^5^^,^^10^

A previous report associated CRP levels and organ failure among critically ill patients, although these patients were not specifically under septic shock.^10^ It considered that CRP was a good marker for organ dysfunction after septic shock had been diagnosed. However, disagreeing with that report, the results presented here, as well as in previously published results,^19^^,^^20^ did not establish any positive correlation between CRP and SOFA.

The present study had some limitations. The diagnosis of infection was based on the presence of positive cultures from blood, urine, catheter or tracheal secretion, or on the presence of a presumed focus of infection in the surgical site. It is known that false-negative cultures are a frequent finding among critically ill patients. Thus, some septic patients may be misdiagnosed as presenting SIRS, instead of sepsis. In order to minimize this source of error, patients diagnosed with SIRS who presented any positive culture during the seven-day observation period were excluded.

## CONCLUSION

CRP is not a good predictor for infection among patients presenting septic shock during the early postoperative period.
